# L‐Theanine Mitigates Aβ1‐42‐Induced Apoptosis in SH‐SY5Y Cells

**DOI:** 10.1002/fsn3.71180

**Published:** 2025-11-11

**Authors:** Yuwei Chen, Zhendong Wang, Jieying Wang, Juan Yang, Ru Ma, Ke Men

**Affiliations:** ^1^ Institute for Research on Health Information and Technology, School of Public Health Xi'an Medical University Xi'an China; ^2^ College of Food Science and Engineering Northwest A&F University Xi'an China

**Keywords:** Alzheimers disease, L‐theanine, neuronal apoptosis, SH‐SY5Y cells

## Abstract

Alzheimer's disease (AD) is a neurodegenerative disorder that significantly impacts the physical and mental health of elderly individuals. Previous research has demonstrated a close association between neurodegenerative diseases and neuronal apoptosis, with cognitive disorders such as AD often exhibiting pathological features, including neuronal apoptosis. In recent years, the application of natural plant molecules for the prevention of AD has emerged as a focal point in food nutrition research. L‐theanine, a unique active compound found in tea, has been shown to possess neuroprotective properties, although its underlying mechanisms remain to be fully elucidated. This study established an in vitro model of AD using SH‐SY5Y cells induced by Amyloid‐beta (1‐42) (Aβ1‐42) to investigate whether L‐theanine can interfere with Aβ1‐42‐induced apoptosis in these cells and to explore its potential mechanisms of action, thereby providing a theoretical foundation for the intervention and prevention of AD. L‐theanine effectively inhibits Aβ1‐42‐induced apoptosis in SH‐SY5Y cells, potentially through the suppression of oxidative stress and preservation of mitochondrial function.

## Graphical abstract

Our research findings indicate that L‐theanine significantly inhibits Aβ1‐42 induced apoptosis in SH‐SY5Y nerve cells, potentially through mechanisms involving the suppression of mitochondrial membrane damage, oxidative stress and the accumulation of extracellular nitrite. These results suggest that L‐theanine is a potential natural substance for intervention in Alzheimer's disease.

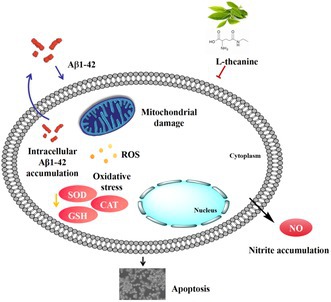

## 1 Introduction

Alzheimer's disease (AD) is a neurodegenerative disorder of the central nervous system characterized by cognitive impairment and memory loss. With the intensification of population aging, AD has become a major global public health issue that humanity needs to confront. The primary pathological features of AD include the accumulation of amyloid‐β protein (Aβ) outside brain neuronal cells, forming senile plaques (SP) in the brain, accompanied by synaptic damage, significant neuronal loss, and cell apoptosis (Guo et al. 2023). The etiology of AD is complex, and current understanding of its pathogenesis remains incomplete. There is no completely effective treatment for AD yet, and most drug therapies can only provide temporary symptomatic relief to slow disease progression and reduce the incidence of cognitive impairment associated with AD.

According to a report by the World Health Organization (WHO), there were 55 million global dementia cases in 2022. It is estimated that this number doubles every 20 years, projected to reach 78 million by 2030 and 139 million by 2050 (Alzheimer's Disease International 2022). The latest epidemiological survey in China shows that among the 15.07 million individuals aged 60 or older with dementia, as many as 9.83 million (65.23%) are affected by AD (Jia et al. 2020). These statistics highlight that AD‐related cognitive impairment is a significant cause of disability and mortality among the elderly, placing a heavy burden on both society and families.

Oxidative stress plays a significant role in the pathogenesis of neurodegenerative diseases. Clinical studies have revealed evidence of oxidative damage to lipids, proteins, and DNA in the central nervous systems of patients with Parkinson's disease (PD) and AD (Jenner 2003). Research indicates that excessive generation of reactive oxygen species (ROS) can interfere with synaptic transmission, leading to a suppression of synaptic efficiency, which is a primary factor in neuronal dysfunction in neurodegenerative diseases. Clinical studies have shown that the “oxidative attack” can be significantly alleviated in most cases once ROS are eliminated by scavengers (Ali et al. 2004). A substantial body of evidence suggests that oxidative stress is a major factor in AD pathogenesis. The accumulation of oxidative stress further exacerbates Aβ deposition and tau protein phosphorylation, disrupting the brain's redox balance. Oxidative modifications of proteins have been observed in the cortex and whole brain of Aβ1‐42 model mice and AD transgenic mouse models (Ghosh et al. 2012).

L‐theanine, an amide compound with the chemical formula C_7_H_14_N_2_O_3_, is structurally similar to glutamate, as shown in Figure S1. Naturally occurring theanine is predominantly in the L‐form (Liu, Wang, et al. 2024; Liu, Tang, et al. 2024). As a natural, water‐soluble analog of glutamate, L‐theanine can cross the blood–brain barrier and act as a competitive antagonist of glutamate receptors. No neurotoxicity has been observed in either in vitro or in vivo studies (Long et al. 2024). Research has demonstrated that L‐theanine possesses neuroprotective properties. For instance, intraperitoneal injection of L‐theanine in mice significantly reduced the volume of infarct caused by middle cerebral artery occlusion without affecting cerebral blood flow or brain temperature, indicating that L‐theanine can cross the blood–brain barrier and provide direct neuroprotective effects (Kakuda 2002). L‐theanine, as a natural and safe substance, has been extensively shown in previous studies to exert neuroprotective benefits. In contrast, traditional pharmacological treatments for AD have shown limited efficacy and are often associated with significant side effects. This study establishes a classical AD cell model using Aβ1‐42 to induce damage in SH‐SY5Y cells and investigates the protective effects and mechanisms of L‐theanine on Aβ1‐42‐induced apoptosis, aiming to provide new insights for the prevention and intervention treatment of AD.

## 2 Materials and Methods

### 2.1 Cell Lines

The human neuroblastoma SH‐SY5Y cell line (Kunming Cell Bank, KCB 2006107YJ) was cultured in T25 flasks and used within three passages. Cells were plated and allowed to recover for 24 h. Differentiation was induced by treatment with 10 μM retinoic acid (RA) in MEM supplemented with 1% FBS for 4 days. The RA‐containing medium was replenished every 48 h. RA was prepared as a 10 mM stock solution in DMSO, stored at −80°C protected from light, and diluted fresh for each medium change. No antibiotics were used during differentiation.

Following differentiation, cells were seeded into 96‐well plates at 5 × 10^3^ cells/well in MEM with 10% FBS and 1% penicillin/streptomycin, and maintained at 37°C in a 5% CO_2_ atmosphere. The cell line was routinely tested and confirmed free of mycoplasma contamination.

### 2.2 Materials and Reagents

L‐theanine (purity ≥ 98%) was purchased from Sigma‐Aldrich (St. Louis, MO, USA; #SMB00395). Commercial assay kits for catalase (CAT; #A007‐1), superoxide dismutase (SOD; #A001‐3), glutathione (GSH; #A006‐2), and malondialdehyde (MDA; #A003‐1) were obtained from Nanjing Jiancheng Bioengineering Institute (Nanjing, China). The Aβ1‐42 ELISA kit (#XL‐EH1658) was purchased from Xinle Biotechnology Co. Ltd. (Shanghai, China). The BCA protein quantification kit (#23225) and ECL luminescence kit (#34580) were supplied by Thermo Scientific (Waltham, MA, USA). RIPA lysis buffer (#P1003B), Annexin V‐FITC/PI apoptosis detection kit (#C1062M), JC‐1 mitochondrial membrane potential assay kit (#C2005), and MTT assay kit (#C0009S) were all obtained from Beyotime Biotechnology (Shanghai, China). A polyvinylidene fluoride (PVDF) membrane (0.45 μm pore size) was procured from Millipore (Billerica, MA, USA). The fluorescent probe DCFH‐DA (#D6883), sulfanilamide (#S9251), N‐(1‐naphthyl)ethylenediamine dihydrochloride (NEDD; #N9125), retinoic acid (RA; #R2625), and dimethyl sulfoxide (DMSO; #D4540) were obtained from Sigma‐Aldrich (St. Louis, MO, USA). The following primary antibodies were used: anti‐GAPDH (Santa Cruz Biotechnology, #SC‐25778), anti‐NQO1 (1:1000 dilution; Abcam, #ab2346), and anti‐HO‐1 (1:1000 dilution; Cell Signaling Technology, #5853).

### 2.3 Cell Culture and Drug Treatment

SH‐SY5Y cells were first cultured in medium containing L‐theanine (0, 10, 25, and 50 μM) for 24 h. After discarding the original medium, the cells were then treated with fresh medium containing 5 μM Aβ1‐42 for 12 h.

### 2.4 Experimental Methods

#### 2.4.1 Observation of Cell Morphology

Following the drug treatment described in Section 2.4.1, cell morphology was assessed using an IX71 inverted fluorescence microscope (Olympus Corporation, Japan) at 200× magnification. Cells were qualitatively evaluated for specific morphological alterations, including neurite outgrowth, cell body shrinkage, nuclear condensation (pyknosis), and membrane integrity. Representative images were captured for documentation.

#### 2.4.2 3‐(4,5‐Dimethylthiazol‐2‐Yl)‐2,5‐Diphenyltetrazolium Bromide (MTT) Assay

The MTT assay (van Meerloo et al. 2011) was utilized to determine the appropriate treatment concentrations of L‐theanine and Aβ1‐42 for subsequent experiments. First, SH‐SY5Y cells were treated with various concentrations of Aβ1‐42 (0, 1.0, 2.5, 5.0, and 10.0 μM) for 24 h to determine its cytotoxic concentration. Based on these results, the time‐dependent effects of Aβ1‐42 exposure were further evaluated to select the optimal incubation period. Concurrently, the cytotoxicity of L‐theanine was assessed by treating SH‐SY5Y cells with concentrations ranging from 0 to 100 μM (0, 10, 25, 50, 80, and 100 μM) for 24 h.

Based on the optimized concentrations of L‐theanine and Aβ1‐42 determined above, cell culture and drug treatment were performed as detailed in Section 2.4.1. Following treatment, the medium in the 96‐well plates was aspirated and replaced with serum‐free medium containing 0.5 mg/mL MTT. The plates were then incubated for 4 h at 37°C under 5% CO_2_ to allow the formation of blue‐purple formazan crystals within the cells. Subsequently, the MTT solution was carefully removed, and the formazan crystals were dissolved in 100 μL of dimethyl sulfoxide (DMSO). The plates were placed on an orbital shaker for 30 min to ensure complete dissolution, after which the absorbance was measured at 490 nm using a multiwell microplate reader (Model 680, American Bole).

$$\begin{array}{c} \text{Cell viability}\;\left(\%\right)\;=\;(\text{Absorbance of treated cells}/\\ \text{Absorbance of control cells})\;\times \;100 \end{array}$$

#### 2.4.3 Annexin V‐FITC/PI Double Staining

Cell culture and drug treatment were performed as described in Section 2.4.1. Apoptosis was detected using an Annexin V‐FITC/PI apoptosis detection kit. Briefly, after cell culture and treatment, the cells were harvested by trypsinization and washed twice with cold PBS (0.15 mol/L, pH 7.2). The cells were centrifuged at 3000 rpm for 5 min, After discarding the supernatant, the pellet was resuspended in 1 × binding buffer at a density of 1.0 × 10^5^ to 1.0 × 10^6^ cells/mL, and incubated with 5 μL of Annexin V‐FITC for 15 min and then with 5 μL of propidium iodide (PI) for 5 min at room temperature in the dark. Finally, the samples were analyzed immediately using a BD Accuri C6 flow cytometer (BD Biosciences), and the data were processed with FCS Express software (De Novo Software).

#### 2.4.4 Intracellular Aβ1‐42 Assayed by ELISA

Following drug treatment, cells were harvested, washed twice with ice‐cold PBS, and lysed through three freeze–thaw cycles in RIPA buffer (50 mM Tris–HCl, pH 7.4, 150 mM NaCl, 1% Triton X‐100) supplemented with 1 × protease/phosphatase inhibitor cocktail. Each cycle involved flash‐freezing in liquid nitrogen for 2 min and thawing in a 37°C water bath for 3 min. The lysates were subsequently centrifuged at 12,000 × *g* for 15 min at 4°C to remove insoluble debris. The resulting supernatants were aliquoted for immediate analysis or stored at −80°C. Prior to ELISA, the total protein concentration of the supernatants was determined using a BCA assay. The samples were then adjusted to 30 μg total protein per 50 μL. The Human Aβ1‐42 ELISA Kit was used according to the manufacturer's protocol, with absorbance measured at 450 nm on the microplate reader. The limit of detection (LOD) is 5 ng/L. Aβ1‐42 concentrations were normalized to total protein content (ng/L protein) and, all measurements were performed in triplicate.

#### 2.4.5 ROS Fluorescence Staining

SH‐SY5Y cells were plated in six‐well plates at a density of 1 × 10^4^ cells/well and cultured overnight in a 37°C, 5% CO_2_ incubator. Following pre‐treatment with L‐theanine (0–50 μM) for 24 h, the medium was replaced with fresh medium containing 5 μM Aβ1‐42 for an additional 12 h. Cells were then incubated with 10 μM H_2_DCFDA (prepared from a 10 mM stock in DMSO, stored at −20°C) in serum‐free medium at 37°C in the dark for 30 min. After washing with PBS, intracellular ROS levels were assessed using an inverted fluorescence microscope. Intracellular ROS levels were quantified using ImageJ by measuring the mean fluorescence intensity (MFI) per cell. Individual cells were outlined as regions of interest (ROI) with background subtraction. For each condition, a minimum of five random fields (≥ 50 cells) were analyzed across three independent biological replicates. Data are expressed as the mean ± SEM.

#### 2.4.6 Antioxidant Marker Levels

Cell culture and drug treatment as described in Section 2.4.1. After treatment, the cells were washed with ice‐cold PBS and lysed in RIPA buffer containing protease inhibitors. The lysates were centrifuged at 12,000 × *g* for 15 min at 4°C, and the supernatants were collected for subsequent assays. CAT activity was determined by monitoring the decomposition of H_2_O_2_ at 240 nm for 1 min in 50 mM phosphate buffer (pH 7.0). GSH levels were measured using a DTNB‐based assay, in which the yellow product was quantified at 412 nm. SOD activity was assessed using a WST‐1 kit by measuring the inhibition of superoxide‐mediated WST‐1 reduction at 450 nm. Lipid peroxidation was evaluated by measuring MDA levels via the thiobarbituric acid reactive substances (TBARS) assay at 532 nm. All measurements were normalized to the total protein content as determined by the BCA assay.

For Western blot analysis, SH‐SY5Y cells were lysed in RIPA buffer containing protease and phosphatase inhibitors. Protein concentrations were determined by the BCA assay. Equal amounts of protein (20 μg per lane) were separated by 10% SDS‐PAGE and transferred to PVDF membranes. Membranes were blocked with 5% non‐fat milk for 1 h at room temperature, then incubated overnight at 4°C with the following primary antibodies: rabbit anti‐NQO1 (1:1000), rabbit anti‐HO‐1 (1:1000), and mouse anti‐GAPDH (1:5000) as a loading control. After washing, the membranes were incubated with HRP‐conjugated secondary antibodies (anti‐rabbit 1:5000; anti‐mouse 1:5000) for 1 h at room temperature. Protein bands were visualized using ECL substrate and quantified with ImageJ software. Relative protein expression levels were normalized to GAPDH and expressed as fold changes compared to the control group. Data are presented as the mean ± SD from three independent experiments.

#### 2.4.7 Intracellular Nitric Oxide Quantification

Cell culture and drug treatment as described in Section 2.4.1, and the concentration of nitrite in cell culture medium was measured by the Griess reagent method (Guevara et al. 1998). Cell culture supernatants were collected after centrifugation. Subsequently, 50 μL of supernatant was mixed with 50 μL of 1% sulfanilamide (in 5% H_3_PO_4_) in a 96‐well plate and incubated at room temperature for 10 min in the dark. Then, 50 μL of 0.1% NEDD was added, followed by incubation at 37°C for 10 min in the dark. Absorbance was measured at 540 nm using a microplate reader.

#### 2.4.8 Mitochondrial Membrane Potential Detection

SH‐SY5Y cells were seeded in 1 × 10^4^ cells/well in 6‐well plates. After drug treatment, the cells were washed three times with PBS and incubated with 5 μg/mL JC‐1 dye in serum‐free medium at 37°C under 5% CO_2_ for 1.5 h in the dark. Following dye loading, the cells were washed twice with warm PBS, and fluorescence was immediately measured using a microplate reader with excitation/emission at 585/590 nm (red, aggregates) and 514/529 nm (green, monomers). The ratio of red to green fluorescence intensity was calculated and normalized to untreated controls (set as 100%) using ImageJ software, with all experiments performed in triplicate and data expressed as the mean ± SD.

### 2.5 Data Statistics and Analysis

All quantitative data are presented as the mean ± standard error of the mean (SEM) from at least three independent experiments. Data were processed using Graphpad Prism software and were considered statistically significant at *p* < 0.05.

## 3 Results

### 3.1 Effects of L‐Theanine on Aβ1‐42‐Induced Morphological Changes in SH‐SY5Y Cells

**FIGURE 1:**
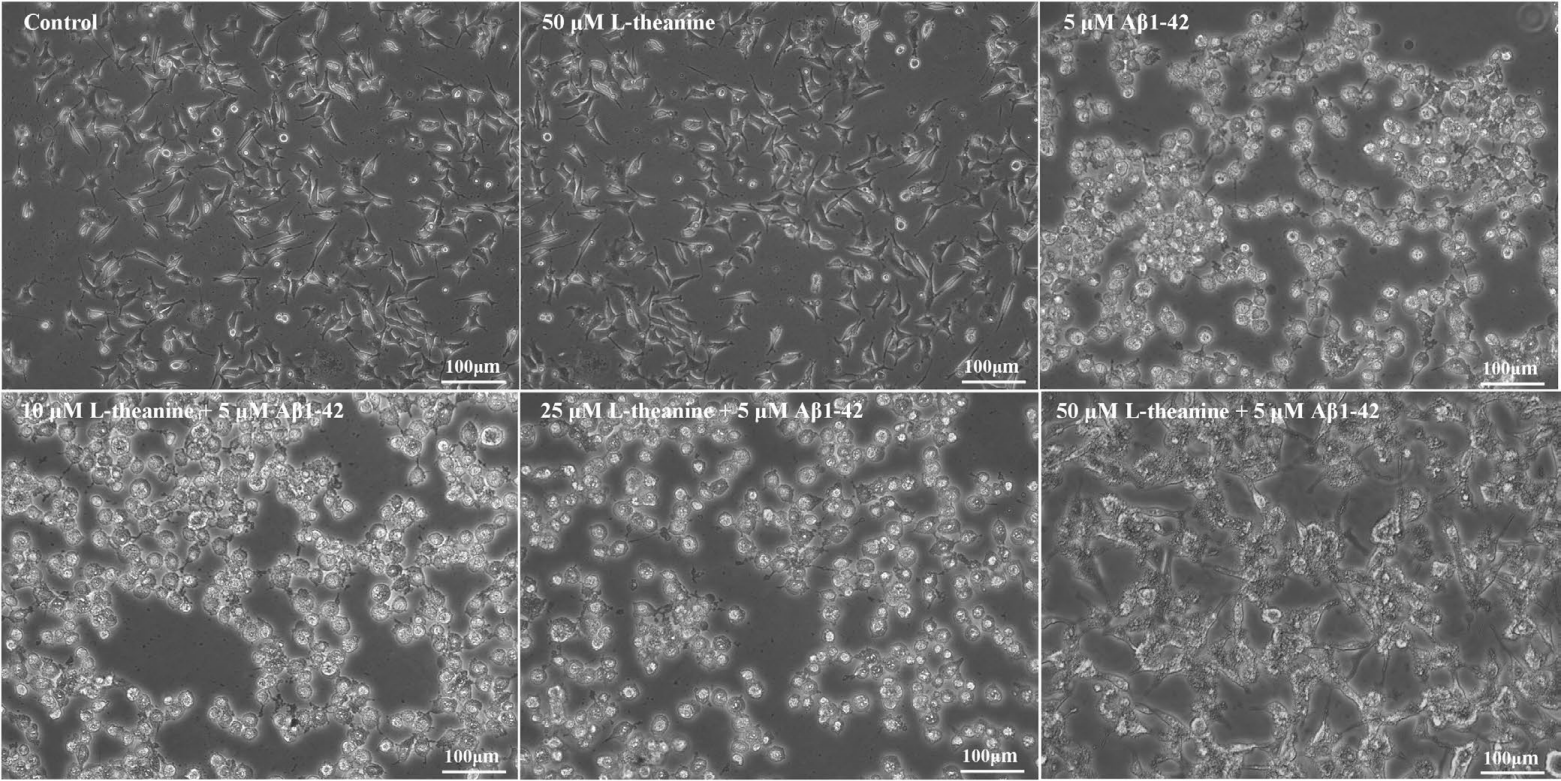
Protective effects of L‐theanine against Aβ1‐42‐induced morphological alterations in SH‐SY5Y cells.

As shown in Figure 1, Aβ1‐42 induction triggered pronounced cytopathological alterations in SH‐SY5Y cells, characterized by significant neurite shortening and nuclear pyknosis. In contrast, cells treated with L‐theanine alone exhibited normal neuronal morphology with intact neurite networks and nuclear structure, indicating that L‐theanine does not intrinsically disrupt neuronal architecture. Notably, pretreatment with L‐theanine significantly attenuated Aβ1‐42‐induced morphological damage, demonstrating preserved neurite integrity and reduced nuclear condensation.

### 3.2 Effects of L‐Theanine on Aβ1‐42‐Induced Viability Changes in SH‐SY5Y Cells

**FIGURE 2:**
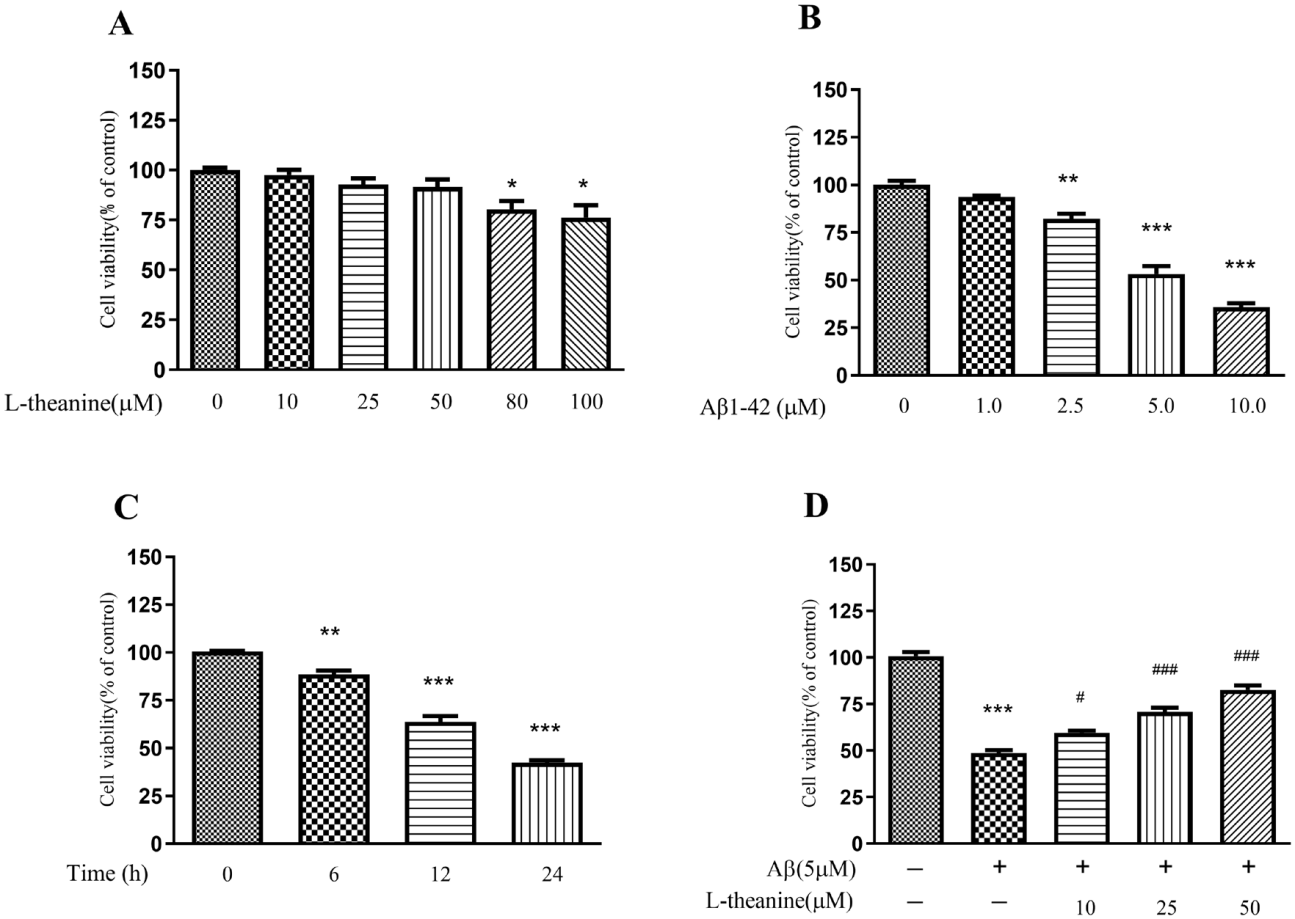
Effects of L‐theanine and Aβ1‐42 on SH‐SY5Y cells' viability assessed by MTT assay. (A) Cells were treated with various concentrations of L‐theanine (0–100 μM) for 24 h. (B) Cells were treated with Aβ1‐42 (0–10.0 μM) for 24 h. (C) Cells were treated with 5 μM Aβ1‐42 for different durations (0–24 h). (D) Cells were pretreated with L‐theanine (0–50 μM) for 24 h, followed by exposure to 5 μM Aβ1‐42 for 12 h. Data are presented as mean ± SEM; **p < 0.01, ***p < 0.001 versus control group; ^#^ *p* < 0.05, ^##^ *p* < 0.01, ^###^ *p* < 0.001 versus Aβ1‐42‐treated group.

SH‐SY5Y cells were incubated with varying concentrations of L‐theanine (0, 10, 25, 50, 80, and 100 μM) for 24 h, followed by assessment of cell viability using the MTT assay (Figure 2A). The results demonstrated that L‐theanine at concentrations below 50 μM did not induce statistically significant reductions in cell viability. However, exposure to 80 μM L‐theanine resulted in a significant 23.64% inhibition of cellular metabolic activity. Based on these findings, the non‐cytotoxic concentration range of 0–50 μM L‐theanine was selected for subsequent cellular experiments.

Exposure to Aβ1‐42 inhibited cell viability in a dose‐dependent manner (Figure 2B). Treatment with 2.5, 5.0, and 10.0 μM Aβ1‐42 significantly reduced cell viability compared to the control group. A concentration of 5 μM Aβ1‐42 was selected for further assays. Subsequently, SH‐SY5Y cells were treated with 5 μM Aβ1‐42 for various durations of 6, 12, and 24 h. Time‐course experiments showed that treatment with 5 μM Aβ1‐42 for 6, 12, and 24 h decreased cell viability in a time‐dependent manner (Figure 2C). Therefore, an injury model was established using 12 h of treatment with 5 μM Aβ1‐42.

The MTT assay further revealed that pretreatment with L‐theanine concentration‐dependently attenuated the Aβ1‐42‐induced reduction in SH‐SY5Y cell viability (Figure 2D).

### 3.3 Effects of L‐Theanine on Aβ1‐42‐Induced Apoptosis in SH‐SY5Y Cells

**FIGURE 3:**
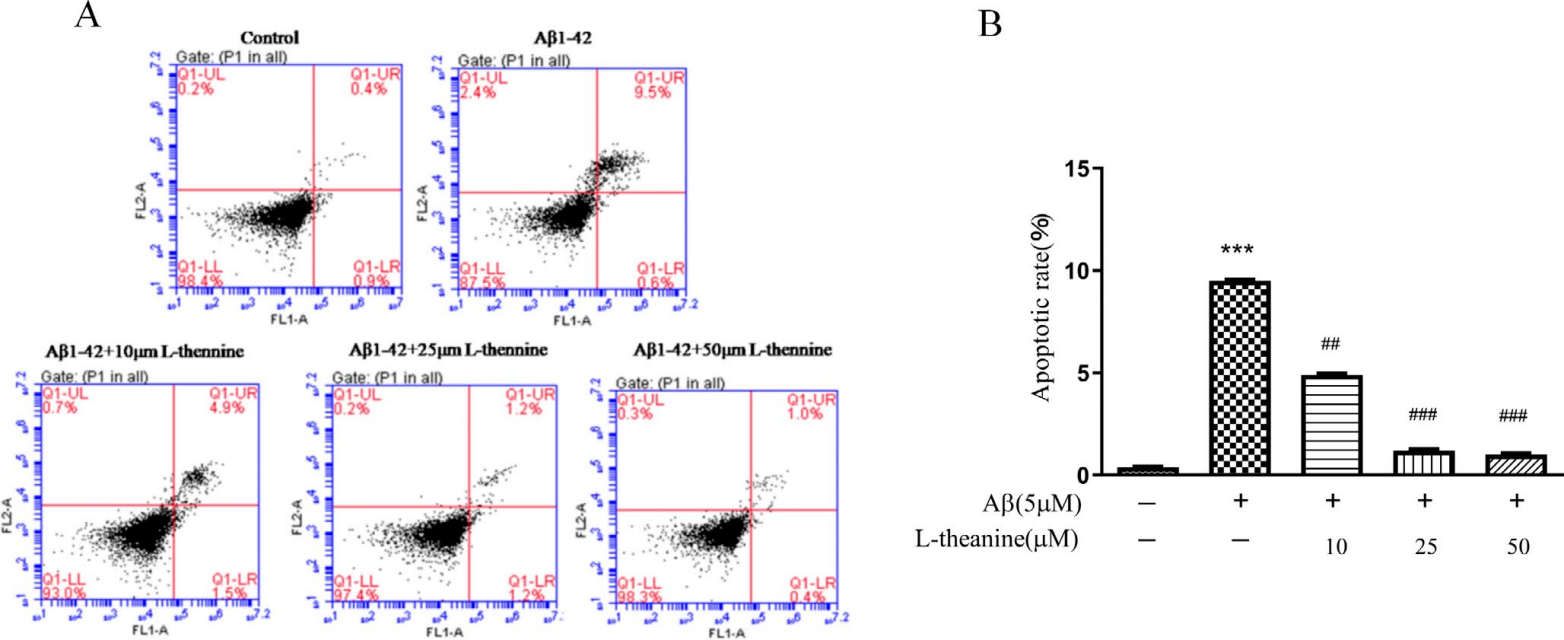
L‐theanine attenuates Aβ‐42‐induced apoptosis in SH‐SY5Y cells. (A) Representative flow cytometry plots. (B) Quantitative analysis of apoptosis. Data are presented as mean ± SEM. ***p < 0.001 versus control group; ^##^ *p* < 0.01, ^###^ *p* < 0.001 versus Aβ1‐42‐treated group.

The apoptotic rates of SH‐SY5Y cells in different treatment groups were assessed using the Annexin V‐FITC/PI method (Figure 3A). The control group showed an apoptosis rate of 0.4%, while the Aβ1‐42‐treated group demonstrated a significant increase in apoptosis (9.5%), indicating Aβ1‐42‐induced neurotoxicity. In contrast, the L‐theanine treatment groups exhibited concentration‐dependent inhibition of apoptosis. Specifically, the 50 μM L‐theanine treatment group exhibited an apoptosis rate of 1% (Figure 3B), representing a significant reduction compared to the Aβ1‐42‐treated group.

### 3.4 Effects of L‐Theanine on Aβ1‐42‐Induced Aβ Accumulation in SH‐SY5Y Cells

**FIGURE 4:**
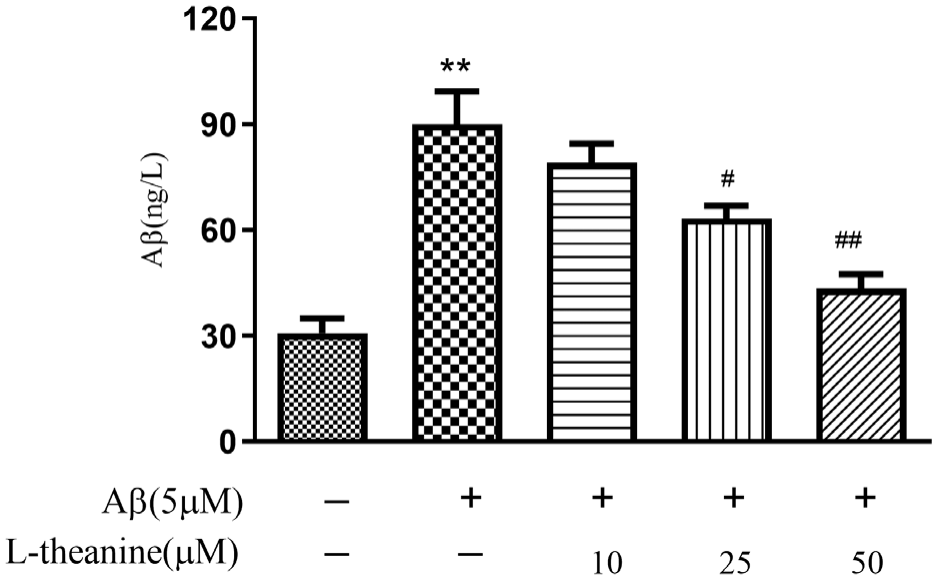
L‐Theanine attenuates Aβ1‐42‐induced intracellular Aβ accumulation in SH‐SY5Y cells. Data are expressed as mean ± SEM. **p < 0.01 versus control group, ^#^ *p* < 0.05, ^##^ *p* < 0.01 versus Aβ1‐42‐treated group.

As shown in Figure 4, compared with the control group, the intracellular Aβ1‐42 content was significantly increased in the Aβ1‐42‐treated group. However, L‐theanine reduced the intracellular Aβ1‐42 levels in a concentration‐dependent manner. These findings suggest that L‐theanine may alleviate the cytotoxic effects by inhibiting the entry of Aβ1‐42 into SH‐SY5Y cells.

### 3.5 Effects of L‐Theanine on Aβ1‐42‐Induced ROS Generation in SH‐SY5Y Cells

**FIGURE 5:**
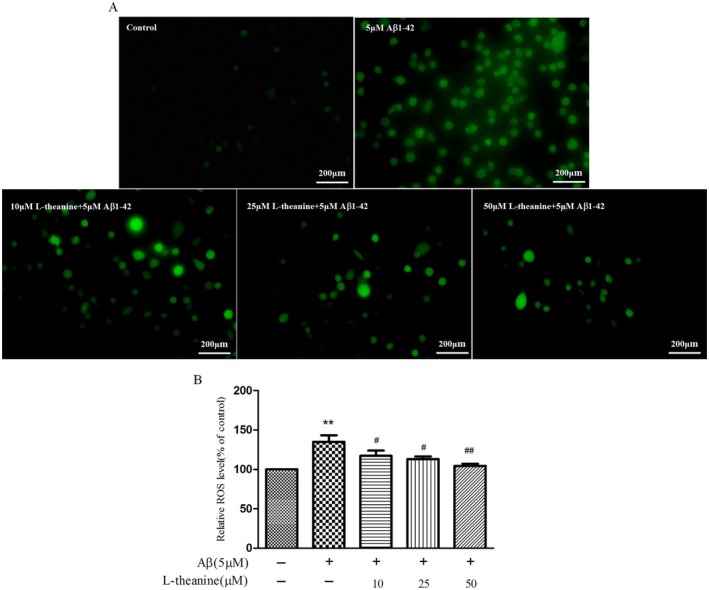
L‐theanine attenuates Aβ1‐42‐induced intracellular ROS generation in SH‐SY5Y cells. (A) Representative fluorescence images of intracellular ROS levels detected by DCFH‐DA staining. (B) Quantitative analysis of ROS fluorescence intensity. Data are presented as mean ± SEM. **p < 0.01 versus control group, ^#^ *p* < 0.05, ^##^ *p* < 0.01 versus Aβ1‐42‐treated group.

As shown in Figure 5, the Aβ1‐42‐treated group exhibited a significant increase in intracellular ROS compared to the control group, as indicated by enhanced green fluorescence. L‐theanine reduced ROS‐associated green fluorescence intensity in a concentration‐dependent manner, thereby decreasing intracellular ROS levels. These findings demonstrate that L‐theanine effectively attenuates Aβ1‐42‐induced oxidative stress in SH‐SY5Y cells.

### 3.6 Effects of L‐Theanine on Aβ1‐42‐Induced Alterations of Antioxidant Markers in SH‐SY5Y Cells

**FIGURE 6:**
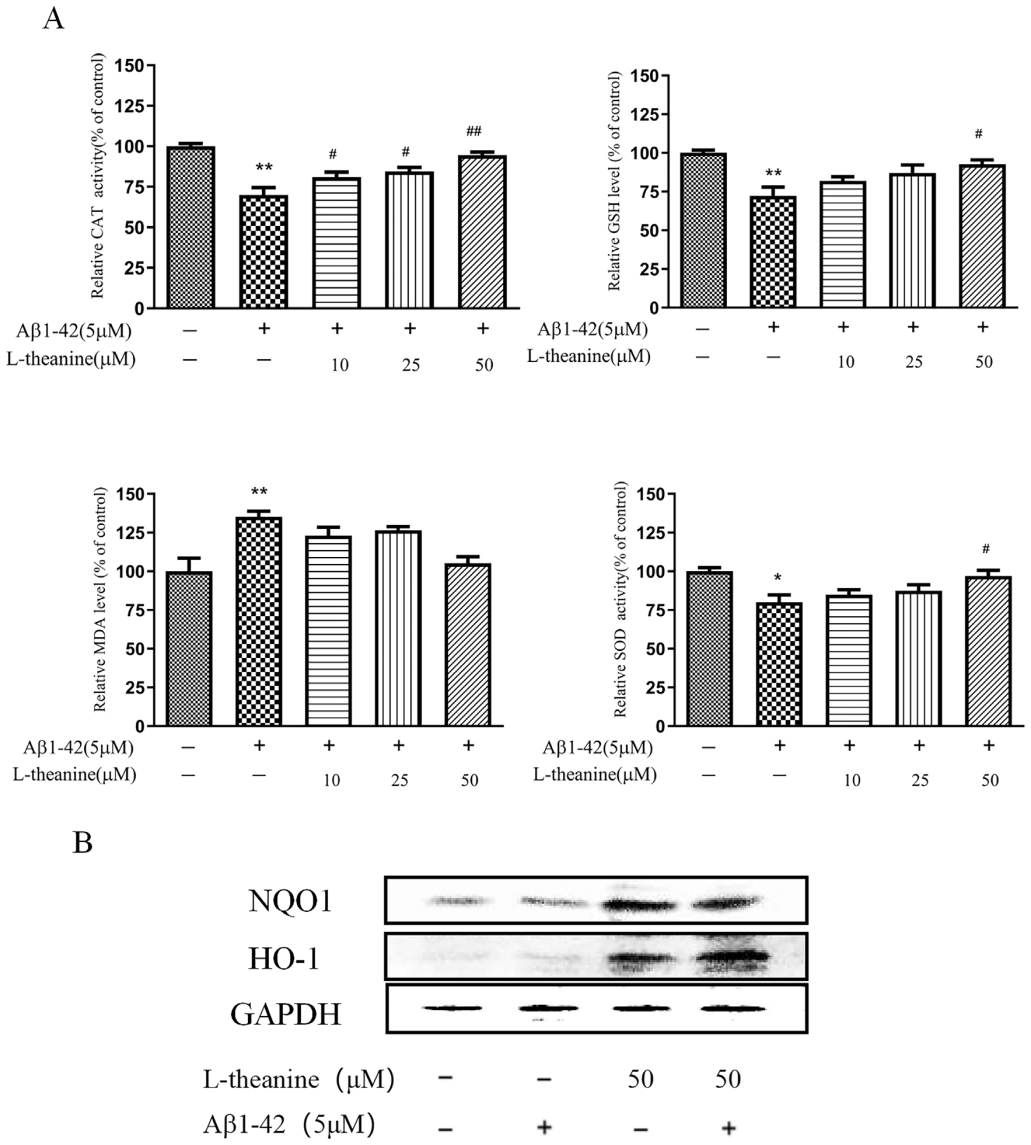
L‐theanine ameliorates Aβ‐42‐induced dysregulation of antioxidant markers in SH‐SY5Y cells. (A) Detection of CAT, GSH, MDA, and SOD levels in Aβ1‐42‐induced SH‐SY5Y cells. (B) Protein expression levels of HO‐1 and NQO1 were detected by western blot. Data are presented as mean ± SEM. *p < 0.05, **p < 0.01 versus control group, ^#^ *p* < 0.05, ^##^ *p* < 0.01 versus Aβ1‐42‐treated group.

After exposure to various drug treatments, the levels of antioxidant markers such as CAT, SOD, GSH, and MDA were evaluated by relevant kits. The experimental results are shown in Figure 6A. Compared with the control group, Aβ1‐42 induction significantly decreased the activities of CAT and SOD as well as the content of GSH, while markedly increasing MDA accumulation. These alterations indicate that Aβ1‐42 disrupts the endogenous antioxidant defense system in SH‐SY5Y cells. Pretreatment with L‐theanine dose‐dependently attenuated Aβ1‐42‐induced antioxidant dysfunction, significantly restoring the activities of SOD and CAT and showing a tendency to recover GSH levels, while slightly reducing MDA accumulation (non‐significant). These findings suggest that L‐theanine exerts protective antioxidant effects against Aβ1‐42‐induced oxidative damage in SH‐SY5Y cells.

To further investigate the regulatory effects of L‐theanine on the antioxidant enzymes heme oxygenase‐1 (HO‐1) and NAD(P)H quinone dehydrogenase 1 (NQO1), we measured their protein expression levels by western blot analysis (Figure 6B). Aβ1‐42 treatment significantly downregulated the expression of both NQO1 and HO‐1 compared with the control group. Notably, pretreatment with 50 μM L‐theanine effectively reversed these changes, upregulating the expression of these enzymes compared with the Aβ1‐42‐treated group.

### 3.7 Effects of L‐Theanine on Aβ1‐42‐Induced Nitrite Production in SH‐SY5Y Cells

**FIGURE 7:**
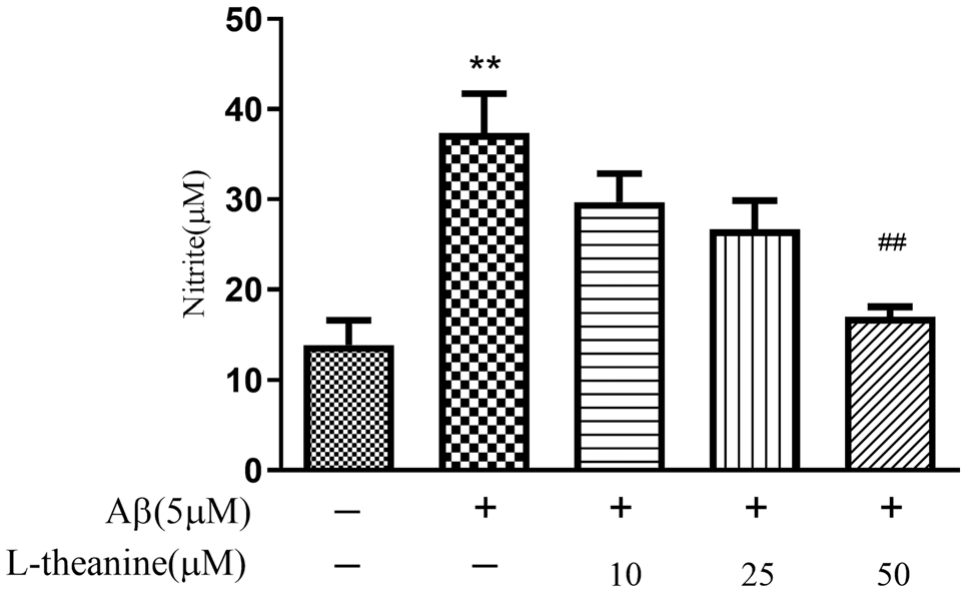
L‐Theanine suppresses Aβ1‐42‐induced nitrite production in SH‐SY5Y cells. Data are presented as mean ± SEM. **p < 0.01 versus control group, ^##^ *p* < 0.01 versus Aβ1‐42‐treated group.

Elevated nitrite reflects sustained NO overproduction, which reacts with superoxide to form peroxynitrite (ONOO^−^), thereby exacerbating oxidative damage (Liu, Wang, et al. 2024; Liu, Tang, et al. 2024; Lv et al. 2025). The extracellular nitrite accumulation of SH‐SY5Y cells was measured using the Griess assay. As shown in Figure 7, treatment with Aβ1‐42 significantly elevated extracellular nitrite levels compared to the control group. In contrast, L‐theanine dose‐dependently reduced Aβ1‐42‐induced nitrite accumulation, with the 50 μM treatment achieving a 50.40% reduction in extracellular nitrite levels compared to the Aβ1‐42‐treated group.

### 3.8 Effects of L‐Theanine on Aβ1‐42‐Induced Mitochondrial Membrane Potential Changes in SH‐SY5Y Cells

**FIGURE 8:**
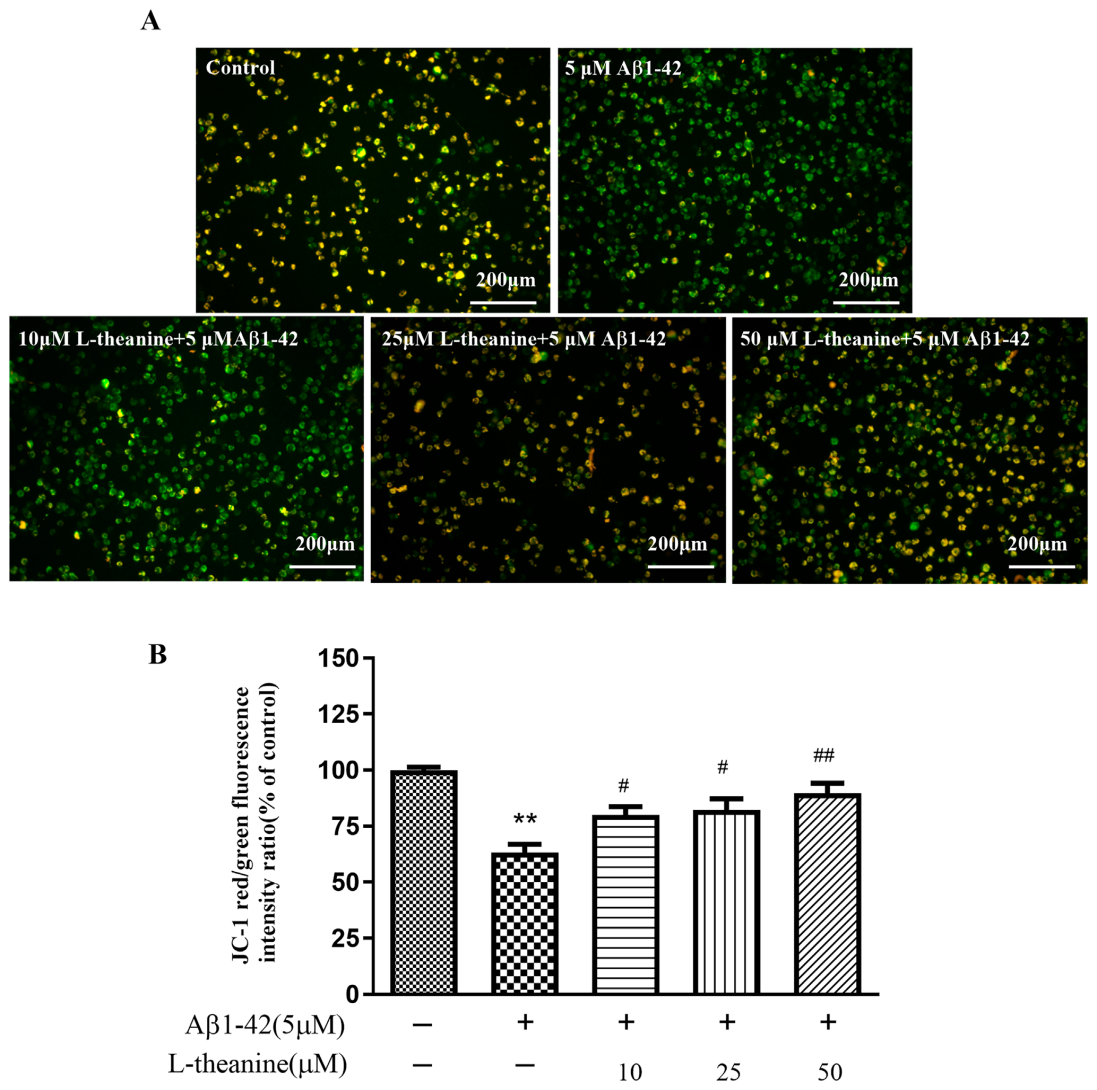
L‐theanine ameliorates Aβ1‐42‐induced mitochondrial impairment in SH‐SY5Y cells. (A) Representative fluorescence images of JC‐1 staining. (B) Quantitative analysis of the red/green fluorescence intensity ratio. Data are presented as mean ± SEM. **p < 0.01 versus control group, ^#^ *p* < 0.05, ^##^ *p* < 0.01 versus Aβ1‐42‐treated group.

Mitochondrial membrane potential was monitored with the JC‐1 assay. As shown in Figure 8A, the Aβ1‐42‐treated group significantly enhanced green fluorescence intensity compared with the control group. L‐theanine treatment resulted in a dose‐dependent increase in red/green fluorescence ratio (Figure 8B), suggesting a protective effect on mitochondrial membrane potential.

## 4 Discussion

This study systematically characterizes the potential protective effects of L‐theanine against Aβ1‐42‐induced neurotoxicity in SH‐SY5Y cells. Key findings demonstrate that L‐theanine: (i) significantly attenuated neuronal apoptosis; (ii) decreased intracellular Aβ1‐42 accumulation; (iii) suppressed oxidative stress through dose‐dependent reduction of nitrite levels and promoted antioxidant markers (CAT, SDO, GSH, HO‐1, and NQO1); and (iv) preserved mitochondrial function. These findings provide critical experimental evidence supporting the application of L‐theanine in AD intervention.

First, this study confirmed that L‐theanine significantly reduced the Aβ1‐42‐induced apoptosis rate in SH‐SY5Y cells. As a key toxic protein in AD pathogenesis, Aβ1‐42 aggregation triggers neuronal apoptosis (Xi et al. 2023). Mechanistically, previous studies have demonstrated that L‐theanine markedly upregulated the expression of the anti‐apoptotic Bcl‐2 protein while downregulating the pro‐apoptotic molecules Bax and caspase‐3. This anti‐apoptotic effect was associated with theanine‐mediated inhibition of MAPK phosphorylation (Kumar et al. 2023), consistent with its previously reported neuroprotective effects in ischemic brain injury and PD models (Ratih et al. 2023). Notably, L‐theanine caused no significant decrease in SH‐SY5Y cell viability, indicating its non‐toxic nature toward neuronal cells. Our flow cytometric analysis revealed distinct apoptotic rates across experimental groups: control group (0.4%) versus Aβ1‐42‐treated group (9.5%). L‐theanine treatment concentration‐dependently inhibited Aβ1‐42‐induced apoptosis, with the 50 μM L‐theanine group showing only a 1% apoptotic rate, a significant reduction compared to the Aβ1‐42‐treated group. These results suggest that L‐theanine may alleviate AD progression through apoptosis inhibition.

The study investigating the effect of L‐theanine on intracellular Aβ1‐42 accumulation in SH‐SY5Y cells induced by Aβ1‐42. The results revealed that the Aβ1‐42‐treated group exhibited significantly higher intracellular Aβ1‐42 levels compared to the control group. Notably, L‐theanine elicited a concentration‐dependent decrease in intracellular Aβ1‐42 accumulation, suggesting its potential role in either inhibiting Aβ production or enhancing its clearance. This finding aligns with cohort population studies highlighting the neuroprotective properties of green tea. Growing evidence in recent years has highlighted the neuroprotective potential of bioactive compounds derived from tea constituents. Active components in green tea enhance vascular perfusion, stimulate hippocampal neurogenesis, and attenuate neuronal apoptosis induced by neurotoxic agents such as free radicals and Aβ (Shibata et al. 2025). Epigallocatechin‐3‐gallate (EGCG) modulates inflammatory pathways implicated in neurodegeneration, potentially delaying pathological progression in neurodegenerative disorders. Furthermore, EGCG reduces tau hyperphosphorylation and aggregation and promotes the non‐amyloidogenic route of APP processing (Valverde‐Salazar et al. 2023). L‐theanine, as a principal bioactive constituent of green tea, potentially shares analogous regulatory mechanisms with other neuroprotective compounds; however, its precise molecular pathways demand systematic elucidation through targeted investigations.

This study demonstrated that L‐theanine concentration‐dependently suppressed intracellular ROS generation in Aβ1‐42‐induced SH‐SY5Y cells, indicating its efficacy in ameliorating oxidative stress. The observed decline in SOD and CAT activities, coupled with GSH depletion, suggested a collapse of the endogenous antioxidant defense system under Aβ1‐42‐induced oxidative stress. This imbalance permits ROS accumulation, leading to lipid peroxidation (as evidenced by elevated MDA) and subsequent neuronal damage. Notably, Aβ1‐42 exposure significantly suppressed the expression of HO‐1 and NQO1—key regulators of oxidative stress and inflammation. L‐theanine treatment effectively reversed this suppression, restoring both enzymes to near‐baseline levels.

The cellular antioxidant defense system comprises enzymatic (e.g., SOD, CAT) and non‐enzymatic (e.g., vitamins C/E, glutathione) components (Chen and Zhong 2014). A bidirectional relationship exists between oxidative stress and Aβ deposition: oxidative stress promotes Aβ generation via ROS‐mediated activation of β‐secretase (BACE1) and γ‐secretase, while Aβ accumulation exacerbates ROS production, establishing a pathological feedback loop (Cheignon et al. 2018). As oxidative stress constitutes a core mechanism in AD pathogenesis, the observed antioxidant properties of L‐theanine align with its documented neuroprotection in diverse neurodegenerative models. Mechanistically, L‐theanine has been shown to mitigate myocardial ischemia–reperfusion injury by suppressing apoptosis and oxidative stress via JAK2/STAT3 signaling (Li et al. 2024). Our findings further substantiate its therapeutic potential in countering Aβ toxicity through enhancement of cellular antioxidant defenses.

L‐theanine additionally attenuated nitrite levels significantly in Aβ1‐42‐induced SH‐SY5Y cells. As a critical mediator of neuroinflammation, excessive NO induces mitochondrial dysfunction and neuronal apoptosis (Nunes and Laranjinha 2021). Building upon these experimental findings, we hypothesize that L‐theanine likely reduces NO production by suppressing inducible nitric oxide synthase (iNOS) expression, thereby mitigating neuroinflammatory cascades.

Quantitative JC‐1 analysis revealed that L‐theanine restored mitochondrial membrane potential in Aβ1‐42‐induced SH‐SY5Y cells. Mitochondrial dysfunction represents a hallmark of AD pathology, and L‐theanine's observed preservation of mitochondrial integrity, potentially via maintaining membrane potential, inhibiting mitochondrial permeability transition pore (mPTP) opening, or modulating energy metabolism, aligns with its documented mitochondrial protective effects in cerebral ischemia models (Xie et al. 2022). A randomized controlled trial demonstrated that 6‐week supplementation with the mitochondria‐targeted antioxidant MitoQ significantly enhanced endothelial function in older adults, as evidenced by improved NO‐mediated endothelium‐dependent dilation (EDD). (Murray et al. 2023).

While this study provides mechanistic insights into L‐theanine's neuroprotective effects, several limitations should be acknowledged. First, the exclusive use of SH‐SY5Y cells, while well‐validated for preliminary AD research, cannot fully recapitulate the complexity of human neuronal networks or the role of non‐neuronal cells (e.g., glia) in AD pathology. Secondly, the lack of in vivo validation in animal models (e.g., APP/PS1 mice) leaves open questions about bioavailability, dose–response relationships, and systemic effects. Further translational development would benefit from: (1) chronic dosing studies in AD rodent models assessing both biomarker modulation (cerebral Aβ, p‐tau) and cognitive outcomes using Morris water maze or novel object recognition tests; (2) bioavailability studies quantifying brain parenchyma concentrations after oral administration; and (3) clinical pilot studies evaluating CSF biomarkers in mild cognitive impairment patients. Importantly, the excellent safety profile of L‐theanine in humans (U.S. Food and Drug Administration [FDA] 2023) significantly lowers barriers for such translational work. These steps are essential to advance L‐theanine from a promising functional ingredient to an evidence‐based neuroprotective agent.

In summary, our findings demonstrate that L‐theanine exerts potent neuroprotection against Aβ1‐42‐induced neuronal apoptosis in SH‐SY5Y cells. This protective effect is mediated through a triad of mechanisms: restoration of endogenous antioxidant defenses, mitigation of mitochondrial dysfunction, and reduction of intracellular Aβ1‐42 accumulation. These findings provide a theoretical foundation for developing natural dietary bioactive compounds targeting neurodegenerative disorders, emphasizing the translational potential of L‐theanine as a functional food ingredient.

## Author Contributions

**Yuwei Chen:** conceptualization (lead), data curation (lead), formal analysis (lead), methodology (lead), project administration (lead), resources (lead), validation (lead), writing – original draft (lead). **Zhendong Wang:** data curation (equal), formal analysis (equal), methodology (equal), software (equal). **Jieying Wang:** formal analysis (equal), methodology (equal), project administration (equal), software (equal). **Juan Yang:** methodology (equal), project administration (equal), visualization (equal). **Ru Ma:** investigation (equal), methodology (equal). **Ke Men:** conceptualization (supporting), funding acquisition (supporting), writing – review and editing (lead).

## Ethics Statement

The authors have nothing to report.

## Conflicts of Interest

The authors declare no conflicts of interest.

## Supporting information


**Figure S1:** Chemical structure of glutamate and L‐theanine.

## Data Availability

No datasets were generated or analyzed during the current study. All conclusions are based on theoretical analysis/review of existing literature.
