# Supplementary figures and images for: L‐Theanine Mitigates Aβ1‐42‐Induced Apoptosis in SH‐SY5Y Cells
Source: Food Sci Nutr. 2025 Nov 11;13(11):e71180. doi: 10.1002/fsn3.71180 (PMC12603780; doi:10.1002/fsn3.71180)

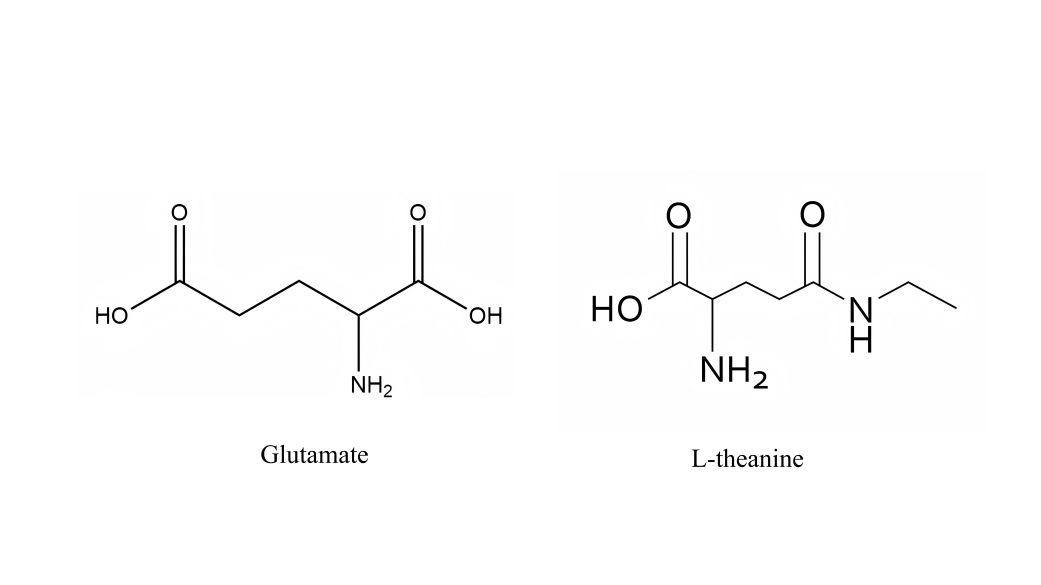


Supplementary Figure S1. Chemical structure of glutamate and L-theanine

Supplement: Supplementary file 1 — Figure S1: Chemical structure of glutamate and L‐theanine. [file FSN3-13-e71180-s001.docx]
